# PSMA expression level predicts differentiated thyroid cancer aggressiveness and patient outcome

**DOI:** 10.1186/s13550-019-0559-9

**Published:** 2019-10-15

**Authors:** Martina Sollini, Luca di Tommaso, Margarita Kirienko, Chiara Piombo, Marco Erreni, Andrea Gerardo Lania, Paola Anna Erba, Lidija Antunovic, Arturo Chiti

**Affiliations:** 1 Department of Biomedical Sciences, Humanitas University, Pieve Emanuele, Italy; 2 Department of Nuclear Medicine, Humanitas Clinical and Research Center - IRCCS, Rozzano, Italy; 3 Department of Pathology, Humanitas Clinical and Research Center – IRCCS, Rozzano, Italy; 4 Department of Advanced Optical Microscopy, Humanitas Clinical and Research Center – IRCCS, Rozzano, Italy; 5 Department of Endocrinology, Humanitas Clinical and Research Center – IRCCS, Rozzano, Italy; 6 Regional Center of Nuclear Medicine, University of Pisa, Pisa, Italy

**Keywords:** Differentiated thyroid cancer, Theragnostic, Glutamate carboxypeptidase II, Biomarkers

## Abstract

**Background:**

Prostate-specific membrane antigen (PSMA) is overexpressed on the endothelial cells of tumor neo-vessels of several solid malignancies, including differentiated thyroid cancer (DTC). We aimed to test the potential role of PSMA as a biomarker for DTC aggressiveness and outcome prediction.

We retrospectively screened all patients who underwent thyroidectomy between 1 January 2010 and 31 December 2017 in our institution. Applying the inclusion (histological diagnosis of thyroid cancer and tissue availability) and exclusion criteria (no clinical or follow-up data or diagnosis of medullary thyroid cancer), a cohort of 59 patients was selected. The monoclonal mouse anti-human PSMA antibody was used to stain tissue sections. A 3-point scale was used to score PSMA positivity: 0–5% expression was considered as negative (score 0), 6–50% as moderately positive (score 1), and 51–100% as highly positive (score 2). A cumulative score (0–10%, 11–79%, and 80–100%) was also explored. Univariate and multivariate logistic regression analyses were performed to predict the presence of distant metastases, chosen as endpoint of aggressiveness. The area under the curve (AUC) was calculated. Cox models were built to predict patient outcome in terms of recurrence, iodine refractoriness, and status at last follow-up, which were calculated using the Kaplan-Meier failure function.

**Results:**

At immunostaining, 12, 25, and 22 patients had scores of 0, 1, and 2, respectively. According to the cumulative score, PSMA expression was ≤ 10% in 17 cases, 11–79% in 31 cases, and ≥ 80% in 11 cases. At multivariate analysis, age, sex, histotype, vascular invasion, T and N parameters, and PSMA positivity were significant predictors of distant metastases. The AUC was 0.92. Recurrence or progression occurred in 19/59 patients. Twelve patients developed radioiodine (RAI) refractoriness, after a median time of 17 months (range 2–32). One patient died of DTC; 46 of the 58 patients alive at last follow-up were disease free. Median DFS was 23 months (range 3–82). The final multivariate model to predict RAI refractoriness included as covariates the stage, high PSMA expression (≥ 80%), and the interaction between moderate PSMA expression (11–79%) and stage.

**Conclusions:**

PSMA, a marker of neovasculature formation expressed by DTC, contributes in the prediction of tumor aggressiveness and patient outcome.

**Electronic supplementary material:**

The online version of this article (10.1186/s13550-019-0559-9) contains supplementary material, which is available to authorized users.

## Background

The incidence of thyroid cancer in Europe in 2018 was estimated to be 11.2 per 100,000 and the disease currently accounts for approximately 1% of all neoplasms, with female affected in 75% of cases [Source: ECIS - European Cancer Information System. From https://ecis.jrc.ec.europa.eu, accessed on 4/04/2019 © European Union, 2019]. While overall survival rates for patients with differentiated thyroid cancer (DTC) exceed 85%, the prognosis of patients with loss of differentiation is poor—survival is limited to 2.5–3.5 years—as a consequence of more aggressive and infiltrative tumor growth and distant metastatic spread. Aggressive DTC also becomes resistant to ablative radioiodine (RAI) treatment, which is the therapy of choice in high-risk DTC. In fact, 5–15% of patients become refractory to RAI.

One requirement for aggressive tumor growth is the development of new blood vessels to provide sufficient blood supply to the tumor. Since advanced thyroid cancer tends to grow aggressively, the tumor neovasculature could serve as a target for imaging as well as therapeutic strategies [1]. Prostate-specific membrane antigen (PSMA) also known as glutamate carboxypeptidase II (GCPII), *N*-acetyl-L-aspartyl-L-glutamate peptidase I (NAALADase I), or *N*-acetyl-aspartyl-glutamate (NAAG) peptidase is one potential target for tumor neovasculature. It is a type II integral membrane protein that is overexpressed in malignancies of the prostate gland and on the cell membrane of endothelial cells of the tumor neovasculature of several solid malignancies [1–3]. Currently, the main indication for ^68^Ga-PSMA PET/CT is relapsing prostate cancer [4]; however, its role in other malignancies, including thyroid cancer, is under evaluation [1, 3, 5–13]. PSMA imaging may be used to visualize tumor lesions and metastases and may provide the rationale for PSMA-targeted radionuclide therapy. This therapeutic option may be extremely promising in DTC since no curative and only a sparse number of palliative treatment options are available for patients with iodine-refractory DTC. The first evidence of PSMA expression in DTC was provided by Verburg et al. [11], who performed ^68^Ga-PSMA PET/CT imaging of a patient with ^131^I-negative scintigraphy who was bearing metastatic poorly differentiated DTC. More recently, immunohistochemical assessment of PSMA expression revealed it to be highly irregularly expressed by thyroid tumors, but not by normal thyroid tissue [12, 14, 15]. PSMA expression has been reported to be significantly associated with tumor size, vascular invasion in follicular carcinoma [12], and poorly or undifferentiated subtypes [14]. Therefore, PSMA expression could be considered as a surrogate endpoint for aggressiveness. However, no data are available on the relationship between PSMA expression and patient outcome. The present study aimed to test the potential role of PSMA as biomarker for DTC aggressiveness (primary objective) and outcome (secondary objective) in terms of earlier recurrence/progression, iodine refractoriness, and presence of disease at last follow-up.

## Methods

### Study design and patient selection

In this retrospective single-center investigation, which was approved by the Ethics Committee of the Humanitas Clinical and Research Center (authorization 02/18, 17 April 2018), we screened all patients who had undergone a thyroidectomy between 01 January 2010 and 31 December 2017. The inclusion criteria were (a) histological diagnosis of thyroid cancer and (b) tissue availability. The exclusion criteria were (a) no clinical or follow-up data and (b) medullary thyroid cancer. From the institutional database, a cohort consisting of 59 patients (mean age 53.12 ± 16.76 years) was selected applying the abovementioned criteria. For all patients, available clinical, biochemical, pathological, and imaging data were retrieved. Demographical data (age and sex) were recorded. Histological data including side of primary tumor, subtype, vascular invasion (absent/present), extrathyroidal invasion, and status of surgical margins (free/involved) were derived from the pathological reports. Gross extrathyroidal invasion defined as extension of macroscopic tumor outside the thyroid gland was assessed on the basis of images and/or clinical reports. All patients were (re)staged according the 8^th^ edition of the American Joint Committee on Cancer (AJCC) staging manual [16]. In all cases, total thyroidectomy or hemithyroidectomy followed by completion thyroidectomy was performed. Seven out of 59 patients were classified as low risk at diagnosis, and accordingly, they did not receive ablative RAI treatment. The main baseline patient characteristics tabulated according to histological subtypes are provided in Additional file 1: Table S1.

### Immunohistochemical staining and analysis

Tissue blocks containing the most representative and well-preserved tumor areas within the formalin-fixed, paraffin-embedded whole tissue sections were selected for immunostaining. Two-micrometer sections were deparaffinized and rehydrated following standard protocol. The monoclonal mouse anti-human PSMA antibody, clone 3E6 (Dako, Agilent Technologies Italia S.p.A.), which recognizes an epitope present in the extracellular portion of the PSMA [17], was used for staining. Methods for immunostaining have been described previously [12, 18]. PSMA expression was evaluated by an experienced pathologist (LDT) on immune-stained whole slides (Olympus, CX 41; × 40 magnification). The 3-point PSMA scale proposed by Bychkov et al. [12] was used to assess and score PSMA positivity. Accordingly, sections with no detectable endothelial PSMA expression and incidental expression in lower than 5% of capillaries were defined as negative (score 0). Sections with PSMA expression in more than 5% of microvessels were defined as positive, and scored as 1 or 2, respectively, according to whether 5–50% or > 50% of microvessels were positive.

More recently, Woythal et al. [18] proposed a different PSMA immunohistochemical 4-point score, derived from neuroendocrine tumors, in prostate cancer. In our analysis, we proposed a modified cumulative score, grouping patients into three classes according to the percentage of PSMA expression: PSMA expression ≤ 10%, PSMA expression between 11 and 79%, and PSMA expression ≥ 80%.

### Endpoint assessment

The primary objective of the study was the prediction of DTC aggressiveness. Among several factors that account for DTC aggressiveness [19, 20], distant metastases at presentation were chosen as the primary endpoint of the present analysis.

The ability of PSMA expression to predict outcome (recurrence/progression, iodine refractoriness, and presence of disease at last follow-up) was tested as the secondary objective. The following endpoints were calculated for this purpose. Disease-free survival (DFS) was computed as the time between thyroidectomy and recurrence (or censored datum) or between ablative RAI and recurrence (or censored datum) in patients who were not and patients who were suitable for ablative RAI, respectively. In patients with metastases at diagnosis, we calculated the progression-free survival instead of DFS. According to the American Thyroid Association Guidelines, patients were defined as iodine refractory when (a) malignant/metastatic tissue never concentrate RAI (no uptake outside the thyroid bed at the first diagnostic or therapeutic whole body scan) or lost the ability to concentrate RAI after previous evidence of RAI-avid disease, (b) RAI concentrated in some lesions but not in others, or (c) metastatic disease progressed despite RAI uptake by lesion(s) [19]. The “refractoriness time” was calculated as the interval between the previous last evidence of RAI-avid disease and the occurrence of one of the abovementioned conditions. Each patient was defined as disease free or not at the last follow-up. Finally, the time between thyroidectomy and last follow-up (or censored datum) or between ablative RAI and last follow-up (or censored datum) was calculated in patients who were not and patients who were suitable for ablative RAI, respectively.

### Statistical analysis

Patient characteristics were summarized in frequency tables. Descriptive statistics were provided for categorical and continuous variables. Statistical analysis was performed using STATA software. PSMA was included as a variable in terms of positivity, percentage of expression, and scores. Demographic data (age and sex), tumor features (side, histological subtype, vascular invasion, status of margins), and stage were tested as variables. Extrathyroidal invasion was not included in the analysis since its definition has changed across different versions of the AJCC staging system. Therefore, data may be not fully comparable unless the revision of histological samples. Univariate logistic regression was used to estimate odds ratios (ORs) and 95% confidence intervals (CIs). Variables satisfying an a priori set criterion of *p* lower than 0.25 were included in the multivariate model to predict distant metastases at presentation. Pathological features including tumor subtype and vascular invasion that have been identified in the literature as strong prognostic factors [19] were a priori included in the analysis. Multicollinearity was checked to test whether a variable included in the multivariate model was closely related to another(s) and, when appropriate, it was/they were removed. The Hosmer-Lemeshow goodness-of-fit test was used to assess the quality of the model. The area under the receiver operating characteristic curve (AUC) was calculated to test the discriminative power of the model.

Outcomes (recurrence, refractoriness, status at last follow-up) were calculated using the Kaplan-Meier failure function. The log-rank test of equality across strata and univariate Cox proportional hazard regression were used to explore whether or not to include the predictor (categorical and continuous variables, respectively) in the final Cox model. Tumor subtype, vascular invasion, and distant metastases, identified in the literature as strong prognostic factors [19], were a priori included in multivariate Cox models in addition to variables satisfying an a priori set criterion of *p* lower than 0.25. Administered RAI activity was tested as confounding factor. Interactions between covariates were also tested. Covariates were omitted in presence of collinearity. Satisfaction of the proportionality assumption was checked by including time-dependent covariates in the model. Only significant variables and significant interaction between covariates were retained in the final model. Model performance was assessed using the Cox-Snell residuals.

A two-sided *p* value of less than 0.05 was considered statistically significant.

## Results

Baseline patient characteristics are reported in Table 1.

**Table 1:** Baseline patients’ characteristics

Characteristics	PSMA expression			Overall
	Negative	Positive		
	Score 0 (*n* = 12)	Score 1 (*n* = 25)	Score 2 (*n* = 22)	*N* = 59
Age				
< 55 years	8	12	13	33
≥ 55 years	4	13	9	26
Sex				
Male	3	6	5	14
Female	9	19	17	45
Histological DTC subtype				
Well differentiated				
Papillary	12	21	16	49
Follicular	0	2	2	4
Poorly differentiated	0	2	4	6
Primary tumor site				
Left lobe	7	8	11	26
Right lobe	2	13	7	22
Left lobe + right lobe	2	2	4	8
Isthmus	0	1	0	1
Left lobe + isthmus	0	1	0	1
Right lobe + isthmus	1	0	0	1
TNM				
T1/T2	6	17	10	33
T3/T4	6	8	12	26
N0	7	19	12	38
N+	5	6	10	21
M0	12	22	16	50
M+	0	3	6	9
Stage				
I/II	12	20	15	47
III/IV	0	5	7	12
Vascular invasion				
No	6	16	7	29
Yes	6	9	15	30
Status of surgical margins				
Free	9	20	16	45
Involved	3	5	6	14
Recurrence				
No	9	19	12	40
Yes	3	6	10	19
Radioiodine refractoriness				
No	10	21	16	47
Yes	2	4	6	12
Status at last follow-up				
No evidence of disease	10	21	15	46
Recurrence/progressive disease or death	2	4	7	13

**Fig. 1:**
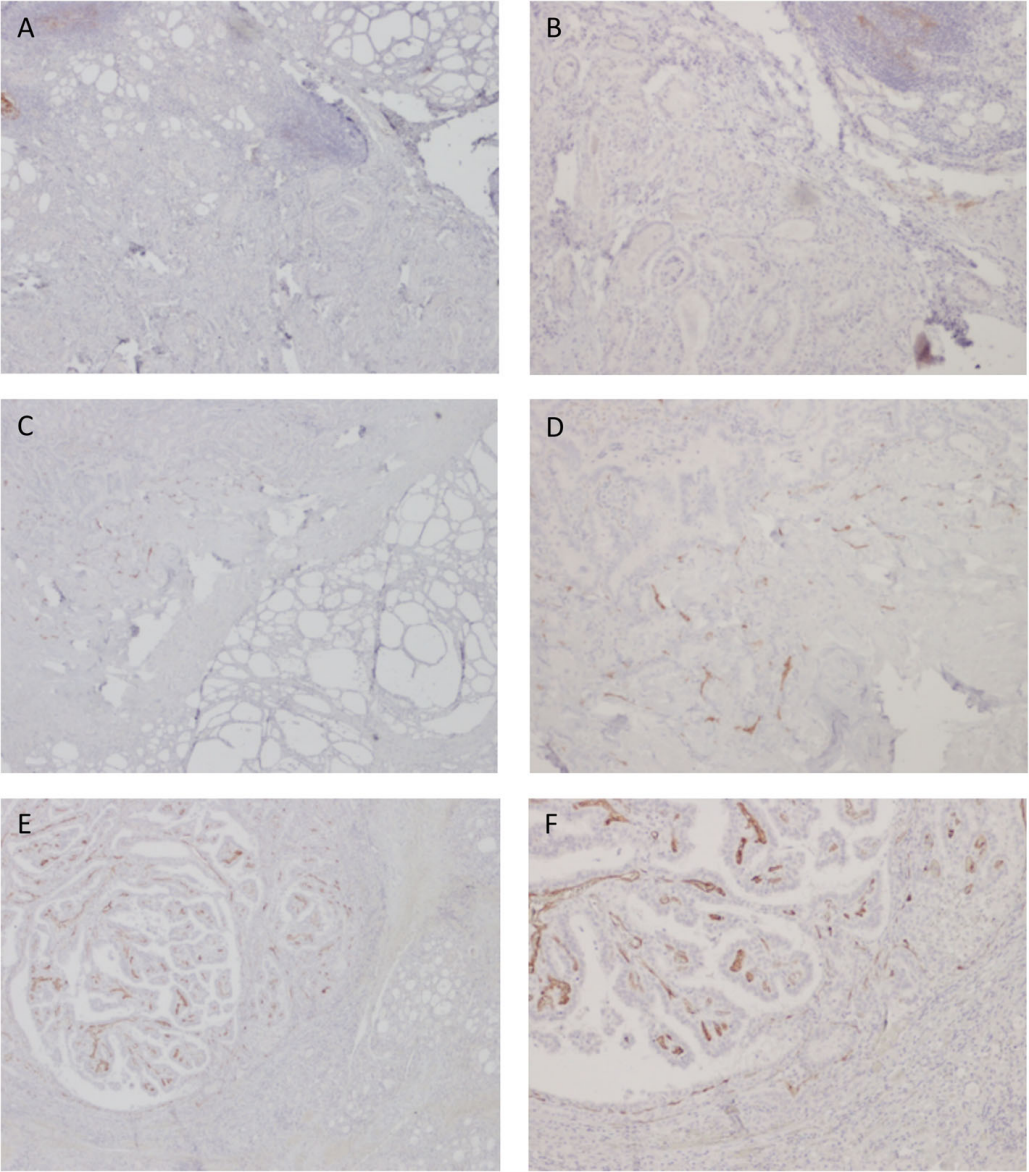
Different patterns of PSMA immunoreactivity in papillary thyroid cancer. **a**, **b** Faint staining is observed in a few endothelial cells, quantified as 1% (PSMA, × 4 and × 20). **c**, **d** Moderate staining is seen in some endothelial cells (PSMA, × 4 and × 20). **e**, **f** Strong stain is present in almost all endothelial cells (PSMA, 4x and 20x)

Immunostaining proved negative in 12/59 patients (20%) and positive in the remaining 47 (80%). Among the 47 positive cases, PSMA expression was scored as moderate (score 1) in 25 (53%) cases and high (score 2) in 22 (47%). Figure 1 shows different patterns of PSMA expression.

According to the cumulative PSMA score, PSMA expression was ≤ 10% in 17of the 59 cases, between 11 and 79% in 31 and ≥ 80% in 11.

### Primary objective: DTC aggressiveness prediction

Distant metastases were histologically confirmed in seven of nine cases. Table 2 summarizes the main characteristics of patients with distant metastases at presentation. Table 3 summarizes results of univariate logistic regression. Among all variables tested, only the side of primary tumor did not satisfy the inclusion criterion for the multivariate analysis. The test for multicollinearity revealed, as expected, a close relationship between age as a continuous and age as a categorical variable (< 55 years and ≥ 55 years) as well as among PSMA variables. We chose to include in the final model age as a categorical variable—as considered in the 8th edition of the AJCC staging system—and PSMA positivity (the PSMA variable that resulted in the lowest *p* value). Parameters of the multivariate logistic regression model are also summarized in Table 3. The model fitted the data well (*p* = 0.62), resulting in an AUC of 0.92.

**Table 2:** Characteristics of patients with distant metastases at presentation

ID	Sex	Age (year)	Side of primary tumor	Histological subtype	Vascular invasion	Status of surgical margins	T parameter	N parameter	M parameter (site)	PSMA expression (%)	Recurrence, time in months	RAI refractoriness, time in months	Status at last follow-up, time in months
#26	F	78	Right	Poorly diff	Yes	Free	pT3	pNx/cN0	pM1 (bone)	40	Yes, 12	Yes, 24 months	No disease-free, 31
#24	F	79	Left	Well diff	Yes	Free	pT2	pN0/cN0	pM1 (bone)	40	Yes, 1	No	No disease-free, 37
#51	M	60	Right	Well diff	No	Free	pT1	pNx/cN0	pM1 (bone)	40	Yes, 27	Yes, 27	No disease-free, 44
#13	F	71	Right	Well diff	Yes	Involved	pT3	pN0/cN0	pM1 (bone)/cM1 (bone, lung)	40	Yes, 6	Yes, 16	No disease-free, 38
#33	M	52	Left	Well diff	Yes	Involved	pT1a	pNx/cN0	pM1 (bone)	40	Yes, 10	Yes, 32	No disease-free, 53
#45	M	78	Right	Well diff	Yes	Involved	pT3	pN0/cN0	pM1 (bone)	70	Yes, 4	Yes, 2	No disease-free, 15
#29	M	47	Right	Poorly diff	Yes	Involved	pT4	pN0/cN0	pM1 (bone)	80	Yes, 5	Yes, 5	No disease-free, 6
#57	F	80	Left	Poorly diff	Yes	Free	pT3	pN1a/cN1 (mediastinum)	cM1 (lung, liver)	80	Yes, 5	Yes, 3	No disease-free, 26
#36	F	76	Right	Well diff	Yes	Free	pT3	pNx/cN0	pM1(bone)	80	Yes, 13	Yes, 5	No disease-free, 87

**Table 3:** Results of the univariate and multivariate logistic regression analysis for prediction of distant metastases at presentation in DTC

Univariate logistic regression				Multivariate logistic regression	
Variable	Odds ratio	95% conf. interval	*p*	Odds ratio	95% conf. interval
Age	1.09	1.02–1.17	0.007	–	–
Age_categorical (< 55 years or ≥ 55 years)	5.71	1.07–30.39	0.041	5.65e+07	n.a.
Sex	0.03	0.07–1.38	0.125	8.70e−09	n.a.
Side of primary tumor	1.01	0.37–2.77	0.979	–	–
Histological subtype	5.75	1.02–32.16	0.046	2.15	0.17–25.94
Vascular invasion	4.10	0.77–21.76	0.097	31.71	0.68–1476.97
Status of margins	1.77	0.38–8.26	0.466	–	–
T parameter	3.00	0.67–13.40	0.150	0.19	0.01–6.75
N parameter	0.18	0.02–1.61	0.128	0.16	0.01–2.32
PSMA positivity	0.23	0.11–0.48	0.000	0.16	0.01–1.94
% PSMA expression	1.02	1.00–1.05	0.042	–	–
PSMA 3-point score	2.13	0.71–6.37	0.174	–	–
Cumulative PSMA score	2.26	0.85–6.05	0.101	–	–

### Secondary objective: DTC outcome prediction

All patients with distant metastases at presentation experienced recurrence or disease progression, as shown in Table 2. An additional ten patients experienced recurrence. Overall, 19 of the 59 patients experienced recurrence (local or lymph node recurrence in 6/19 cases; distant metastases in 8/19 cases) or disease progression (5/19 cases) at a median interval of 11 months (range 3–36). Median DFS was of 23 months (range 3–82). Twelve patients developed RAI refractoriness with a median time of 17 months (range 2–32). Forty-six of the 58 patients alive at last follow-up were disease free; one patient had died from disease-related causes. The median duration of follow-up was 41 months (range 3–168).

The significance of univariate analysis related to outcomes is summarized in Table 4.

**Table 4:** Results (*p* values) of univariate analysis in respect of outcomes

Variable	Recurrence	RAI refractoriness	Status at last follow-up
Age	0.036*	0.009*	0.020*
Sex	0.696	0.147*	0.051*
Side of primary tumor	0.208*	0.041*	0.377
Histological subtype	0.039*	0.029*	<<0.001*
Vascular invasion	0.127*	0.067*	0.168*
Status of surgical margins	0.021*	0.060*	0.419
T parameter	0.473	0.018*	0.118*
N parameter	0.893	0.621	0.542
M parameter	<< 0.001*	<< 0.001*	<< 0.001*
Stage	<< 0.001*	<< 0.001*	<< 0.001*
Administered RAI activity	<< 0.001*	<< 0.001*	0.001*
PSMA positivity	0.497	0.681	0.394
% PSMA expression	0.066*	0.241*	0.130*
PSMA 3-point score	0.451	0.917	0.680
PSMA cumulative score	0.192*	0.213*	0.230*

*Selected for multivariate Cox model

**Fig. 2:**
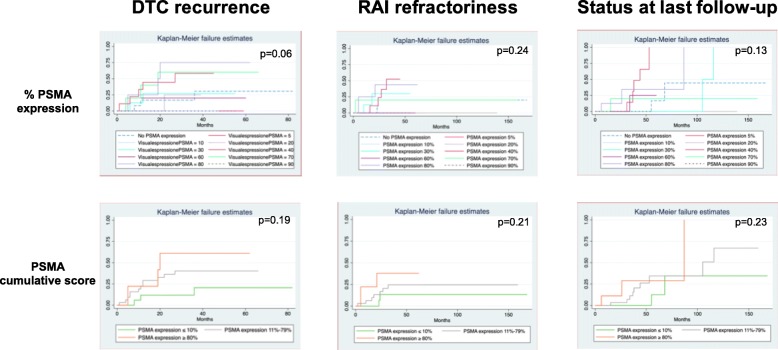
Kaplan-Meier curves and univariate analysis results in relation to PSMA immunoreactivity and outcomes

Figure 2 shows Kaplan-Meier curves and univariate analysis results in relation to PSMA covariables tested as predictors. Additional file 1: Figures S1–S3 show Kaplan-Meier curves and univariate analysis results for clinical-pathological covariables tested as predictors. The multivariate model for prediction of recurrence included the interaction between the right side of the primary tumor and distant metastases, the status of surgical margins, and metastases at presentation as covariates.

The final multivariate model for prediction of RAI refractoriness included the interaction between low-to-high PSMA expression (11–79%) and stage, the stage, and very high PSMA expression (≥ 80%) as covariates.

**Fig. 3:**
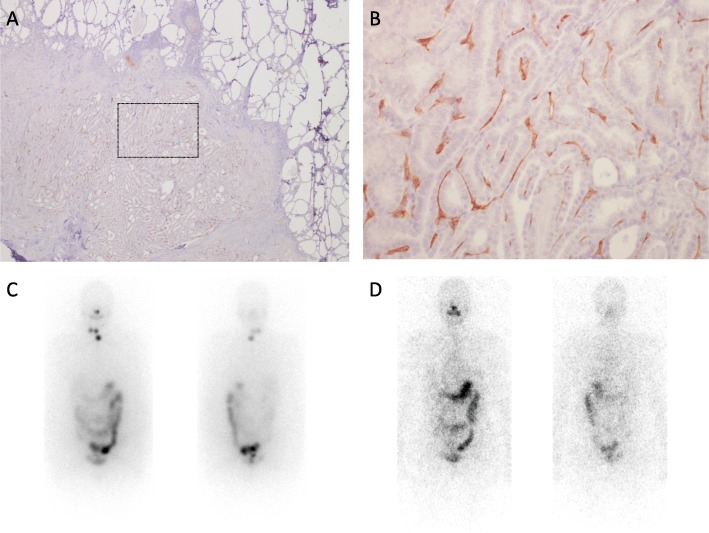
Example of PSMA staining in a 45-year-old female diagnosed with a papillary thyroid cancer (presence of vascular invasion, free surgical margins, pT3mpN1bcM0 – stage I). Panoramic view (**a**, × 2) shows diffuse PSMA staining which involves endothelial cells almost completely (**b**, × 20) and was quantified as 90%. Planar images (**c**) obtained after radioiodine (RAI) treatment (2960 MBq) show uptake in the thyroid bed and in right cervical lymph nodes. At last follow-up (56 months after surgery), the patient was disease free (thyroglobulin 0.2 ng/mL), as also confirmed by diagnostic planar images (**d**) obtained after RAI administration (185 MBq)

**Fig. 4:**
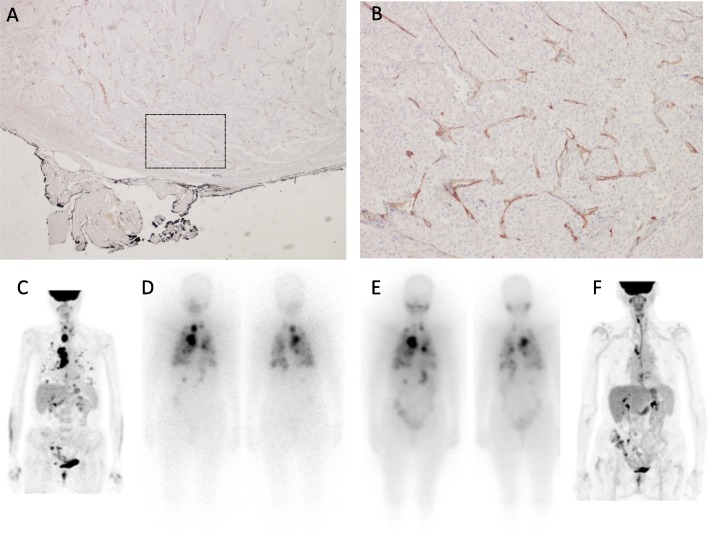
Example of PSMA staining in an 80-year-old female diagnosed with a poorly differentiated thyroid cancer (presence of vascular invasion, free surgical margins, pT3mpN1acM1 – stage IVb). Panoramic view (**a**, × 2) shows the insular pattern of growth highlighted by PSMA staining which involves endothelial cells almost completely (**b**, × 20) and was quantified as 80%. Staging PET/CT image (**c**) shows [^18^F]FDG uptake in the left lobe of thyroid, cervical, and mediastinal lymph nodes, lungs, and liver. Diagnostic whole-body scan (185 MBq) obtained 3 months after thyroidectomy shows several foci of radioiodine (RAI) uptake in the mediastinum, lungs, and liver (**d**). Images (**e**) obtained after RAI treatment (5550 MBq) show uptake in mediastinum, lungs (fewer lesions than on the diagnostic scan), and liver. Accordingly, the patient was defined as RAI refractory. Clinical evidence of disease progression occurred 5 months after treatment (thyroglobulin 2864 ng/mL) as confirmed by PET/CT images showing the appearance of new lung lesions (**f**). At last follow-up (26 months after RAI treatment), the patient presented rapidly clinical progressive disease and palliative therapies were commenced

Figures 3 and 4 show examples of PSMA expression in two patients who differed in terms of histological subtype, stage, and outcome.

Sex, histological subtype, and their interaction as well as the interaction between sex and vascular invasion were included in the final Cox model to predict status at last follow-up.

**Fig. 5:**
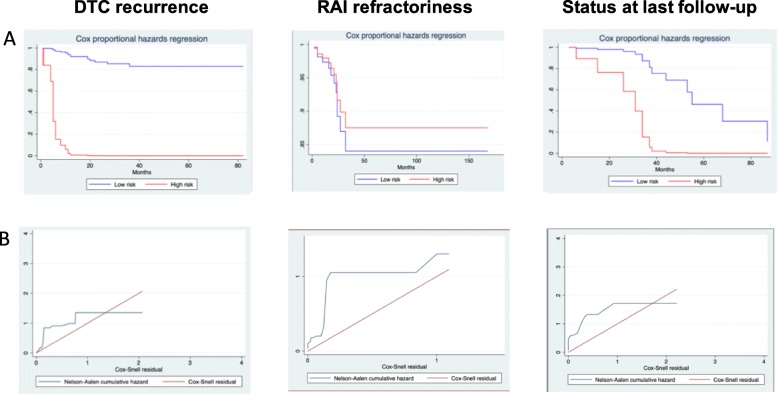
Kaplan-Meier curves of the final Cox models (**a**) and performance of the final models assessed by Cox-Snell residuals (**b**)

The results of the final models are shown in Table 5. Kaplan-Meier curves and models’ performance are illustrated in Fig. 5.

**Table 5:** Multivariate Cox models to predict outcomes

Covariables	Hazard ratio	95% CI	*p*
Recurrence prediction			
Metastases	72.33	12.58–415.65	<< 0.001
Surgical margins	5.06	1.75–14.60	0.003
Metastases-right side of primary tumor^#^	0.18	0.03–0.88	0.035
RAI refractoriness prediction			
Stage	5.34e+09	1.42e+09 to 2.00e+10	<< 0.001
PSMA expression ≥ 80%	3.77e−09	7.18e−10 to 1.98e−08	<< 0.001
PSMA expression 11–79%-stage^#^	1.28e−09	–	–
Status at last follow-up prediction			
Sex	85.26	10.48 to 693.52	<< 0.001
Histological subtype	7.98e−10	2.34e−10 to 2.73e−09	<< 0.001
Sex-histological subtype^#^	7.64e+08	–	–
Sex-vascular invasion^#^	0.01	0.00–0.09	<< 0.001

*CI* confidence interval

^#^Interaction

## Discussion

Our results confirm that DTC expresses PSMA. Moreover, a strong association emerged between PSMA expression and DTC aggressiveness supporting further investigations. In our population, the percentage of cases that expressed PSMA was higher than that reported in the literature (80% versus 50–60%) [12, 14]. However, this finding may be the result of variation in selection criteria among studies. We recruited only cases who had been surgically treated and followed up in our institution for whom a surgical sample of the primary tumor, clinical, and follow-up data was available. These criteria may have resulted in a selection bias, potentially impacting on study findings. In fact, in our cohort only 12% of patients were classified as low risk at diagnosis. Tumors such as microcarcinoma and low-risk DTC are expected to express lower PSMA levels than high-risk DTC. Moreover, since they do not require additional treatment unless their risk changes over time [19], it is more likely that lower-risk patients will be referred to local centers rather than highly specialized institutions such as ours.

In our cohort, elderly patients with poorly differentiated thyroid cancer who presented an advanced stage with distant metastases had the worst prognosis, confirming literature data [19]. As expected, risk of failure was higher in PSMA-positive cases than in PSMA-negative ones, even though statistical significance was not reached. Currently, DTC patients are classified as low, intermediate, or high risk and managed accordingly [19]. Common risk factors include histological characteristics of aggressiveness, extrathyroidal extension, vascular invasion, number and size of involved lymph nodes, and distant metastases [19]. However, none of the available risk stratification approaches is really effective in predicting DTC aggressiveness and recurrence [21]. Since patients with metastatic disease have a poor prognosis, identifying predictors of disease progression and potential new therapeutic targets is critical. In recent years, thanks to the expansion of knowledge on the molecular, genetic, and epigenetic aspects of thyroid cancer, research has aimed to specifically profile tumors with more aggressive behavior and/or resistance to therapy [22]. Immune tumor microenvironment [23, 24], epithelial-mesenchymal transition [25–28], and some peculiar features including *BRAF*^*V600E*^ mutation [29], *TERT* promoter mutations [30, 31], and transcriptomic signatures [32, 33] have been described as predictors of aggressive DTC. Moreover, several serum or tissue miRNAs (either upregulated or downregulated) have been reported to be involved in DTC initiation, progression, and aggressiveness [34, 35]. However, none of these potential biomarkers are currently used in clinical practice. Firstly, prognostic biomarkers should be tested in large populations and their role validated. Secondly, as mentioned previously, the prevalence of metastatic, resistant, or aggressive DTC is relatively low, and an extensive evaluation of molecular and genetic profiles in all DTC patients is probably ineffective as well as too expensive. In this regard, PSMA is promising since immunohistochemistry is a procedure currently used to assess tumor biological profile, similar to HER2 status assessment in breast cancer. The identification of PSMA expression in primary thyroid cancer is easily accessible, inexpensive, and potentially effective; therefore, it represents a candidate biomarker to stratify patients at risk of recurrence.

As expected, poorly differentiated thyroid cancer was associated with an unfavorable final status at last follow-up (Table 5). Poorly differentiated thyroid cancer presents an aggressive behavior—fewer than half of patients survive at 10 years [36]—accompanied by a host of the typical features of thyroid differentiation [19].

The 3-point score [12] used to rate PSMA expression resulted almost clinically meaningless in our population. The cumulative score proposed in the present study adopted the partition applied for PSMA assessment in neuroendocrine tumors [18] but with redefined categories. In summary, our score merged negative cases with those characterized by very low expression (≤ 10%,), grouped together low-to-high PSMA expression (11–79%,), and distinguished patients presenting very high PSMA expression (≥ 80%). This is in line with Her2/neu status evaluation, where negative or mild expression is considered not to be significant. Conversely, high PSMA expression is related to a remarkable neovasculature formation and, therefore, is potentially associated with higher aggressiveness. In line with this speculation, our results showed a higher risk of failure in patients with very high PSMA expression (≥ 80%) than with low-to-high (11–79%) or absent/very low PSMA expression (≤ 10%), even if statistical significance was not reached. Interestingly, a very high PSMA expression and an advanced stage (III/IV) and its interaction with a low-to-high PSMA expression were the covariates retained in the final model to predict RAI refractoriness. Up to 35% of patients with DTC have metastatic cancer [37]. For some patients with metastatic DTC (up to 35%), remarkable results can be obtained by RAI. However, only two thirds of patients with metastases show substantial RAI uptake. RAI refractoriness is more frequent in older patients, in those with large metastases, in poorly differentiated thyroid cancer, and in tumors with high [^18^F]FDG uptake on PET/CT [38]. However, as for recurrence, none of these features is effective in predicting RAI refractoriness. The identification of patients who will develop RAI refractoriness would be of great clinical value since RAI-refractory patients have a 10-year survival rate < 10% with a mean life expectancy of 3–5 years [39]. Our results suggested that stage and PSMA expression could be used to predict RAI refractoriness at diagnosis, potentially impacting on patient management and finally improving outcome. Therefore, our findings pave the way for further investigations to explore whether thyroid cancer microvasculature may be a target for PSMA-directed theragnostic (imaging and treatment), especially in iodine-refractory and aggressive high-grade thyroid carcinomas.

## Limitations

Our study presents some limitations. The retrospective design of the study and the criteria used for patients selection possibly affected our results. The exact value of each thyroglobulin determination was missing for many metastatic patients with a high disease burden. In most of these cases, the lab reported that the thyroglobulin was above a certain value (e.g., > 1000 or 3000 ng/mL). Missing data precluded calculation of the thyroglobulin doubling time and its correlation with PSMA expression. Another limitation of our study was the lack of minimum follow-up time as a criterion for patient selection. Accordingly, in patients with a short follow-up, we assumed that DTC was not aggressive whereas we cannot know whether disease recurred after patients were censored. In fact, in most cases, DTC is a long-lasting disease with an indolent course that can manifest its aggressiveness even 5–10 years after diagnosis. Moreover, as mentioned above, the prevalence of aggressive DTC is quite low. Therefore, future studies will require larger sample sizes in addition to longer follow-up. The relatively small sample size and, additionally, the low incidence of events registered in our cohort were probably the main factors that underpowered the prognostic role of PSMA.

## Conclusions

PSMA, a marker of neovasculature formation expressed by DTC, contributed to contribute to the prediction of tumor aggressiveness and holds promise for outcome prediction. Accordingly, PSMA —easily accessible, inexpensive, and potentially effective especially in predicting RAI refractoriness—represents an elegant candidate biomarker in DTC. Further studies should elucidate the role of PSMA in risk stratification as well as the value of PSMA-based PET imaging and therapeutic applications with beta and alpha emitters.

## Clinical relevance

Up to 35% of patients with DTC have metastatic cancer. For some patients with metastatic DTC, remarkable results can be obtained by RAI. However, only two third of patients with metastases show substantial RAI uptake, and only 42% of them achieve a cure. RAI-refractory patients have a 10-year survival rate < 10% with a mean life expectancy of 3–5 years. Our findings pave the way for further investigations to explore whether thyroid cancer microvasculature may be an effective target for PSMA-directed theragnostic (imaging and treatment), especially in iodine-refractory and aggressive high-grade thyroid carcinomas potentially impacting on patient management and ultimately improving outcome.

## Additional file

Additional file 1: **Figure S1.** Kaplan-Meier curves and univariate analysis results for clinical variables tested as covariates to predict recurrence. **Figure S2.** Kaplan-Meier curves and univariate analysis results for clinical variables tested as covariates to predict radioiodine refractoriness. **Figure S3.** Kaplan-Meier curves and univariate analysis results for clinical variables tested as covariates to predict status at last follow-up. **Table S1.** Main baseline patient characteristics according to histological subtypes. (DOCX 971 kb)

## Data Availability

The datasets used and/or analyzed during the current study are available from the corresponding author on reasonable request.

## Abbreviations

AJCC: American Joint Committee on Cancer
AUC: Area under the receiver operating characteristic curve
CIs: Confidence intervals
DFS: Disease-free survival
DTC: Differentiated thyroid cancer
GCPII: Glutamate carboxypeptidase II
NAAG: *N*-acetyl-aspartyl- glutamate
NAALADase I: *N*-acetyl-L-aspartyl- L-glutamate peptidase I
OR: Odds ratio
PSMA: Prostate-specific membrane antigen
RAI: Radioiodine
WBS: Whole body scan
[^18^F]FDG 2-Deoxy-2-[18F]: fluoroglucose
