# Supplementary material for: PSMA expression level predicts differentiated thyroid cancer aggressiveness and patient outcome
Source: EJNMMI Res. 2019 Oct 15;9:93. doi: 10.1186/s13550-019-0559-9 (PMC6794333; doi:10.1186/s13550-019-0559-9)

**PSMA expression level predicts differentiated thyroid cancer aggressiveness and patient outcome**

**Martina Sollini^1,2^, Luca di Tommaso^1,3^, Margarita Kirienko^1^, Chiara Piombo^3^, Marco Erreni^4^, Andrea Gerardo Lania^1,5^, Paola Anna Erba^6^, Lidija Antunovic^2^, Arturo Chiti^1,2^**

1 Department of Biomedical Sciences - Humanitas University, Pieve Emanuele, Italy

2 Nuclear Medicine - Humanitas Clinical and Research Center - IRCCS, Rozzano (Milano), Italy

3 Pathology - Humanitas Clinical and Research Center - IRCCS, Rozzano (Milano), Italy

4 Advanced Optical Microscopy, Humanitas Clinical and Research Center - IRCCS, Rozzano (Milano), Italy

5 Endocrinology - Humanitas Clinical and Research Center - IRCCS, Rozzano (Milano), Italy

6 Regional Center of Nuclear Medicine - University of Pisa (Pisa), Italy

**Acknowledgements**

We thank Olimpia Alice Manzardo and Katia Marzo for their support in patient selection; the Endocrinology Unit for close collaboration in patient management, treatment and follow-up; the Departments of Oncologic Surgery and Nuclear Medicine for close collaboration in patient treatment; and Paola Magnoni, Livia Saltarin, and Pasquale de Nittis for imaging patients during the follow-up.

**Corresponding author**

Martina Sollini, M.D., PhD

Department of Biomedical Sciences, Humanitas University

Via Rita Levi Montalcini 4, 20090 Pieve Emanuele (Milano) Italy

Tel +39 02 8224 6621

Fax +39 02 8224 6693

Email: martina.sollini@cancercenter.humanitas.it

Additional file 1

Table S1: Main baseline patient characteristics according to histological subtypes

| Characteristics | Well-differentiated thyroid cancer | | Poorly differentiated thyroid cancer (n=6) |
| --- | --- | --- | --- |
|  | Papillary (n=49) | Follicular (n=4) |  |
| Age | | | |
| <55 years | 32 | 0 | 1 |
| ≥55 years | 17 | 4 | 5 |
| Sex | | | |
| Male | 12 | 1 | 1 |
| Female | 37 | 3 | 5 |
| Primary tumor site | | | |
| Left lobe | 23 | 0 | 3 |
| Right lobe | 15 | 4 | 3 |
| Left lobe + right lobe | 8 | 0 | 0 |
| Isthmus | 1 | 0 | 0 |
| Left lobe + isthmus | 1 | 0 | 0 |
| Right lobe + isthmus | 1 | 0 | 0 |
| TNM | | | |
| T1/T2 | 32 | 1 | 0 |
| T3/T4 | 17 | 3 | 6 |
| N0 | 29 | 4 | 5 |
| N+ | 20 | 0 | 1 |
| M0 | 46 | 1 | 3 |
| M+ | 3 | 3 | 3 |
| Stage | | | |
| I/II | 46 | 1 | 0 |
| III/IV | 3 | 3 | 6 |
| Vascular invasion | | | |
| No | 28 | 1 | 0 |
| Yes | 21 | 3 | 6 |
| Status of surgical margins | | | |
| Free | 41 | 2 | 2 |
| Involved | 8 | 2 | 4 |
| Recurrence | | | |
| No | 37 | 1 | 2 |
| Yes | 12 | 3 | 4 |
| Radioiodine refractoriness | | | |
| No | 43 | 1 | 3 |
| Yes | 6 | 3 | 3 |
| Status at last follow-up | | | |
| No evidence of disease | 42 | 1 | 3 |
| Recurrence/progressive disease or death | 7 | 3 | 3 |

**Figure S1. Kaplan-Meier curves and univariate analysis results for clinical variables tested as covariates to predict recurrence**


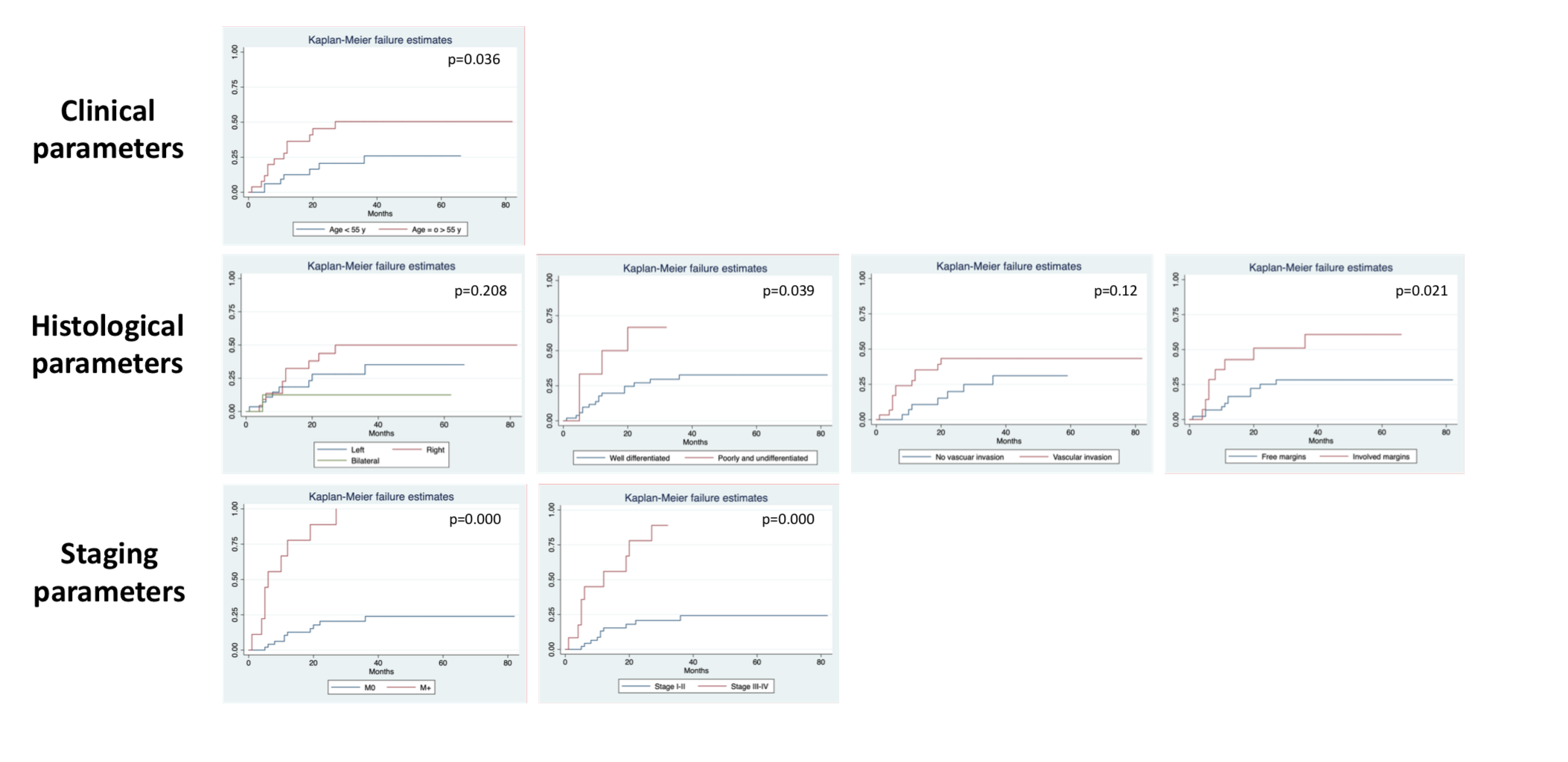


**Figure S2. Kaplan-Meier curves and univariate analysis results for clinical variables tested as covariates to predict radioiodine refractoriness**


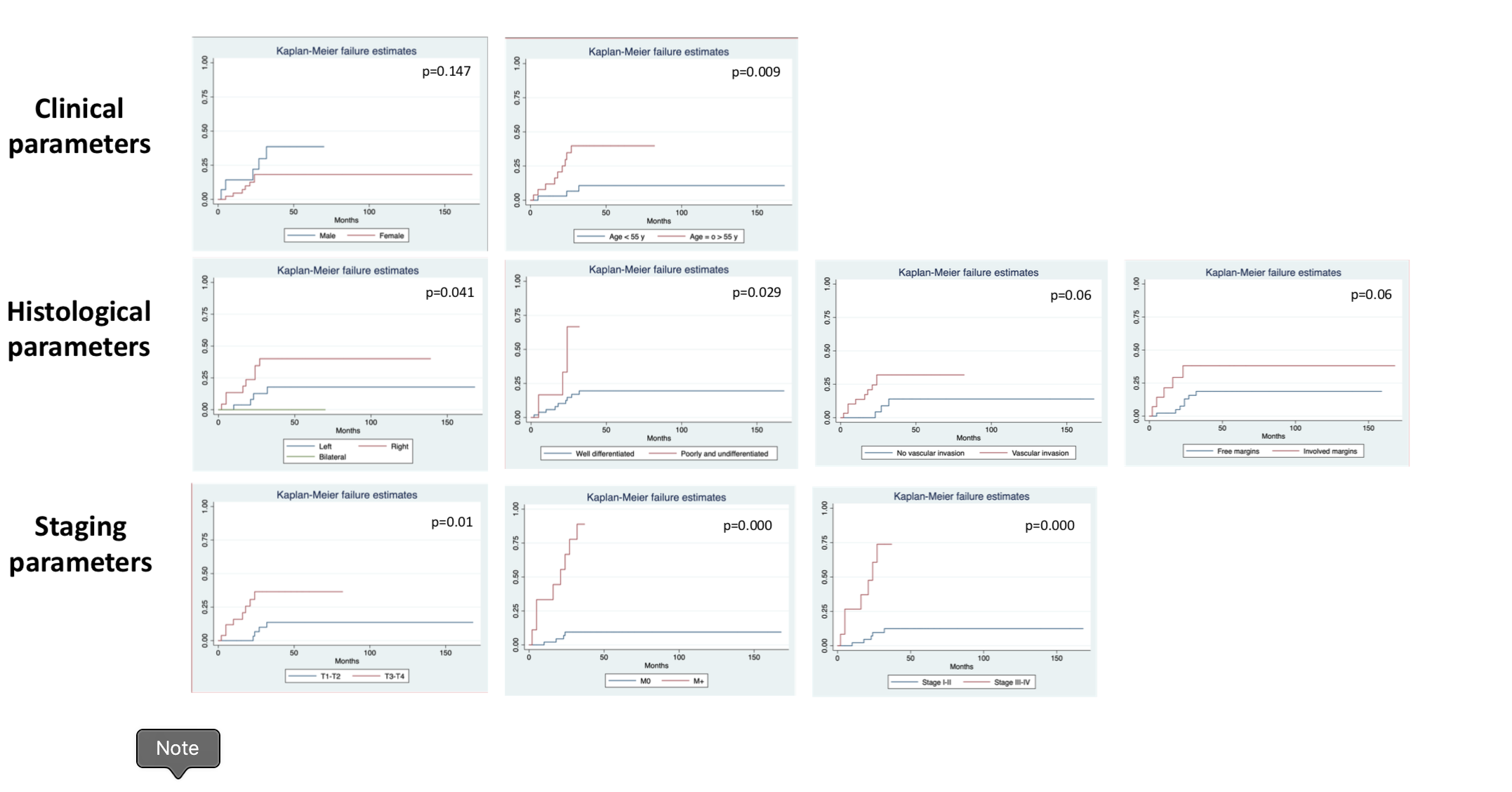


**Figure S3. Kaplan-Meier curves and univariate analysis results for clinical variables tested as covariates to predict status at last follow-up**


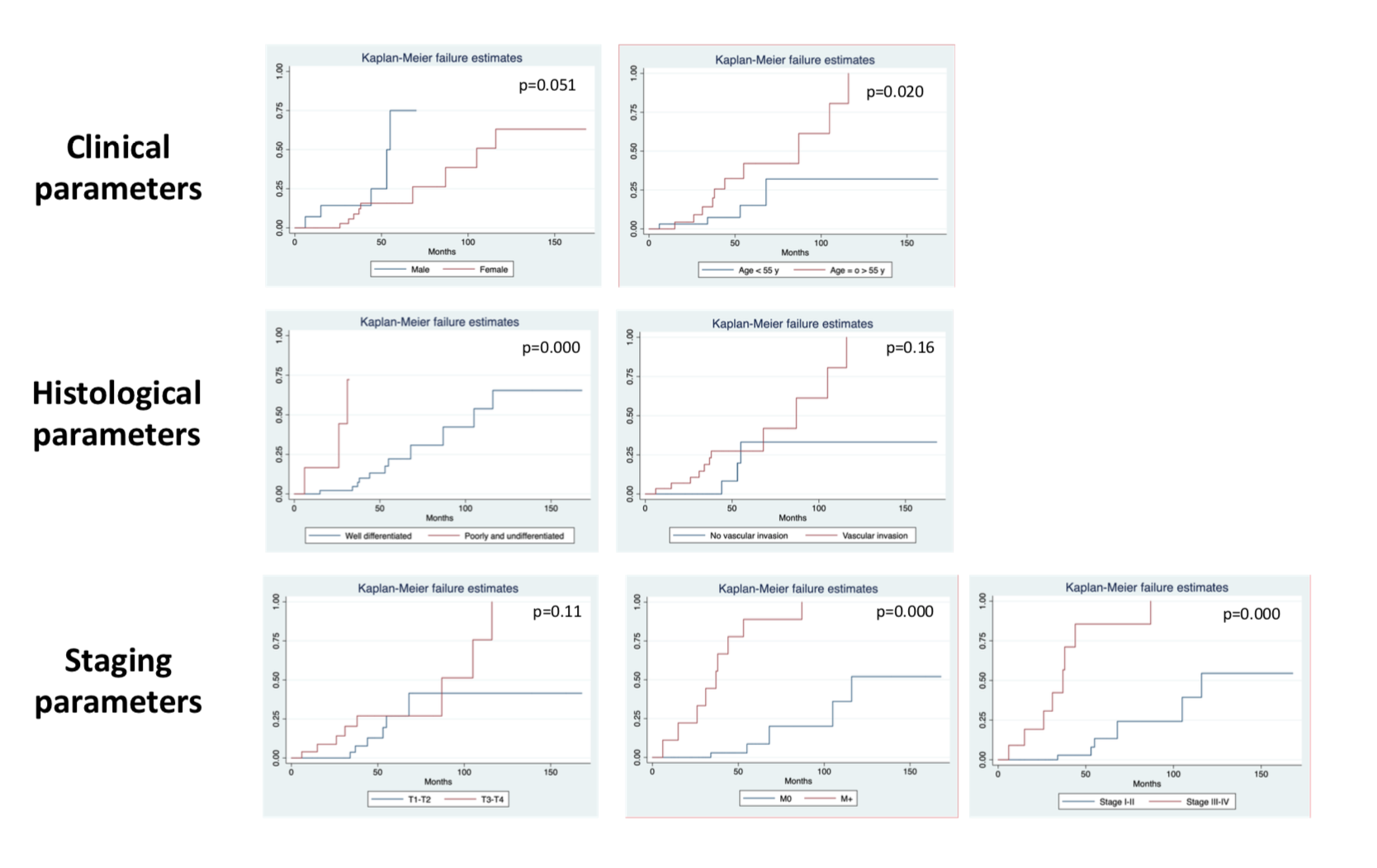

Supplement: Supplementary file 1 — Figure S1. Kaplan-Meier curves and univariate analysis results for clinical variables tested as covariates to predict recurrence. Figure S2. Kaplan-Meier curves and univariate analysis results for clinical variables tested as covariates to predict radioiodine refractoriness. Figure S3. Kaplan-Meier curves and univariate analysis results for clinical variables tested as covariates to predict status at last follow-up. Table S1. Main baseline patient characteristics according to histological subtypes. (DOCX 971 kb) [file 13550_2019_559_MOESM1_ESM.docx]
